# Supplementary material for: Neural mechanisms for the attention-mediated propagation of conceptual information in the human brain
Source: PLoS Biol. 2025 Mar 28;23(3):e3003018. doi: 10.1371/journal.pbio.3003018 (PMC12135929; doi:10.1371/journal.pbio.3003018)
Supplement: Attachment — Submitted filename: Acunzo et al - round 1 revision response to reviewers.docx [file pbio.3003018.s003.docx]

**Response to reviewers**

Neural mechanisms for the attentional propagation of conceptual information in the human brain

Acunzo, Grignolio & Hickey

Reviewer #1: The authors present interesting data from an innovative study that relates variation of the N2pc component to representation of categorical/conceptual information. Across two experiments, elegant multivariate analyses are employed to reveal how attention influences the propagation of conceptual information in the brain. Experiment 1 is a pure EEG experiment, whereas Experiment 2 extends a similar logic to an EEG-fMRI experiment. The results are rich and interesting, overall shedding light on how attention guides the abstraction of information in the human brain.

I enjoyed reading this manuscript. As best I can tell, the methods are innovative and rigorous (one small comments on thresholding below), and the results are systematic and interesting. I think the manuscript can make an important contribution both on the content level, as well as methodologically. I do have a couple of comments, however, which I would ask the authors to address.

1) The authors acknowledge that variation in the N2pc could reflect various processes (including misallocation of attention), but the question of the source of this variation is not discussed (or explored) in great detail. Specifically, I was wondering in how far results may be in particular driven by full misallocation in the sense of having attended and responded to the wrong item (of course there are other ways of making a mistake, but since the responses expected for the target and distractor were opposite, it seems likely that wrong responses often simply reflect responding to the wrong stimulus), which would likely have a strong influence on the results that would go beyond "increasingly robust attentional selection of the target", which implies something more gradual. In this context, I am not fully sure whether incorrect trials were excluded (sorry, if I missed this information). So, just to be sure, were they?

Our thanks to R1 for the insightful evaluation of the MS.

Incorrect trials were excluded from analysis. We have revised to clarify and emphasize this point in the new MS (in the “Data Acquisition and Preprocessing” sections of methods for each of the two experiments). To perhaps preempt a follow up question, the ability to confidently exclude data from single trials in MRI analysis is provided by use of the GLMsingle toolbox. We model the HRF function within each correct and incorrect trial, such that variance stemming from incorrect performance is partitioned specifically to incorrect trials. When we average the beta weights observed in correct trials, we can be confident that the values associated with correct performance are minimally impacted by hemodynamic variance created in incorrect trials.

The reviewer expresses interest in results when participants fully misallocate attention and respond to the wrong stimulus. Unfortunately, accuracy in the experiments is high, at around 90%, with individual participants who achieve above this mean value. This leaves few trials for an examination of neural dynamics that precede incorrect performance.

That said, the results do suggest that stronger selection of the distractor is associated with degraded processing of the target. This emerges in the opposite directionality of the relationship between N2pc and category information in E1 for target-elicited and distractor-elicited N2pc, and in a similar pattern in analysis of the ventromedial prefrontal cluster in E2. When selection of the target is robust - as reflected in target-elicited N2pc - category information for the target emerges more strongly. But when selection of the distractor occurs - as reflected in distractor-elicited N2pc - there is less information about the target category. In the revised manuscript we more clearly identify this pattern as an expression of the misallocation of attention (p.10).

2) Fig. 2: I think the EEG data recorded inside the scanner looks really good; to further illustrate this, I think it would be good to add a topo plot to panel D, just like the one in panel A.

Response Figure 1 – Topography of the right-minus-left difference for target lateral conditions in Experiment 2. As in the topography for Experiment 1, which is illustrated in Manuscript Figure 2a, the N2pc expresses as a posterior negativity in the left hemisphere but a positivity in the right hemisphere due to the underlying subtraction.

To be frank, the left-minus-right-target topography from E2 is messy. This in part reflects the reduced data quality, with fewer subjects and fewer trials per subject. But, more than this, it is a product of scanner-induced artefacts.

Posterior electrodes are relatively fixed in space, with the weight of the head keeping them in place. Anterior electrodes move more due to subtle rotation of the head, movement of the anterior scalp, vibration, and pulse, and we find substantially more residual muscle / movement / cardioballistic noise on these channels. In the grand average, this happens to vary between target-left and target-right trials.

This conditional difference could be a product of random chance. But we think it might have been an unintended consequence of how we presented stimuli. We had a slight issue with the MRI coil and EEG equipment occluding the presentation of stimuli. To circumvent this, the stimuli array was not always presented directly in front of the participant head position, with the central fixation mark appearing up to ~5 degrees of visual angle lateral to head position.

We were not particularly concerned with this during data collection, as the contralateral and ipsilateral signals used to identify N2pc are defined relative to fixation, not head orientation. But in hindsight we believe that this may have introduced a difference between target-left and target-right conditions. Participants may have been more prone to make eye movements in one condition versus the other, or the difference may have impacted the likelihood or amplitude of sub-saccadic eye movements (ie. microsaccades). Under normal circumstances we expect this would have had minimal impact on the results, particularly after artefact correction. But in the scanner, we believe these differences in oculomotor effects translated into rather substantive conditional differences in muscle / eye rotation / ballistic artifacts in the EEG that are not entirely removed through correction.

This makes the left-minus-right topography illustrated in Response Figure 1 confusing. The topography clearly expresses the N2pc in the posterior lateral foci, but also has prominent effects at electrodes over the temples (likely muscular in origin) and a prominent anterior effect (likely muscular / oculomotor). These do not have a clear stimulus-evoked pattern in the results and we don’t believe that they reflect brain activity. However, this non-standard topography of the N2pc may confuse readers more than it helps clarify the results.

Then, you could also more clearly indicate that the sensor plots on the left and on the right rely on slightly different channels (2 sets of three electrodes for the EEG-only experiment, and only 1 electrode on each side for the EEG-fMRI dataset, if I understood correctly).

The use of different electrodes for the two experiments reflects the increased sensor density in E1, where 128 channels are recorded rather than 64. This allows us to measure the N2pc at sets of 3 electrodes where the component is known to emerge with maximal amplitude, rather than the single electrodes employed in E2.

Tell us if the indication of electrode location is too subtle; we can increase the size of the markers, or of the topography altogether, if the current figure is ambiguous or insufficient.

3) I was not sure why the current set-up yielded a distractor N2pc, rather than a distractor positivity. It is quite possible that I am misunderstanding something, and/or it may relate back to my first question, but it may be useful to clarify.

There are two issues here. The first is methodological: the stimuli arrays we employ are not entirely balanced in terms of sensory energy. As discussed at more length in our response to R3, point 3, the morphed placeholder images structurally differ from the complete recognizable images, most obviously in spatial frequency. As a result, the lateral placeholder consistently elicited a contralateral signal that differed from that elicited by the complete image, and the ipsilateral and contralateral signals accordingly diverge from about 100 ms post-stimulus.

This is hard to avoid. To our knowledge, there is no principled way to generate a heterogenous set of objects that are not real-world objects but have the same low-level features as real-world objects. In any case, the situation means that the N2pc emerges in the context of an ongoing lateral signal, and that makes it difficult to distinguish the contribution of Nt and Pd subcomponents. That is, the Pd is defined as a positive polarity contralateral signal when the contralateral signal otherwise has a baseline value of zero. If the contralateral baseline signal has unknown non-zero value, the Pd may emerge as a reduction of ongoing contralateral negative voltage. There is a hint of this in our data; the distractor-elicited N2pc is of low amplitude and is immediately followed by a consistent positive polarity lateral signal. We think this reflects the underlying Pd, which emerges only after the non-N2pc lateral negativity ‘wears out’.

We do not discuss this at length in the paper because it is confusing and not directly relevant to the results we observe. That is, we test the possibility that Pd correlates with distractor category information in the paper. We extract the amplitude of the lateral ERP response in the latency of N2pc / Pd, and compare this to distractor category information, with the idea that a more positive lateral response might predict less category information. These analyses are briefly described in the paper, but results are ambiguous and far from reliable (though in the predicted direction). This effect, if it exists, is too small for us to detect with this design and sample. Given that there are ultimately no effects associated with Pd, we feel it would be confusing to discuss the component in the paper.

The second issue is that our search displays are simple, with two vibrant naturalistic images, and participants are not under time pressure and are relatively slow to respond. In this circumstance, the mis-deployment of attention does not have dire consequences; participants can recover and redeploy attention to the target before it disappears (or degrades from iconic memory). We expect that participants are more likely to ‘look around’ in this circumstance, deploying attention to the complete object during search for the cued annulus. They are thus less likely to suppress the distractor, and more likely to select it. This is conjecture, and not an issue that we discuss in the paper.

4) It would also be good to clarify a bit how the MR and EEG data were synchronized during acquisition. It is stated that the EEG (and VCG) data was acquired at MR clock speed (5 kHz), but I believe that the MR clock is much faster. Without any further synchronization (see below), also the choice of number of slices vis-à-vis the TR could have been a problem (since a slice duration results that is not divisible by the EEG sampling rate, which is not recommended, I think). However, you also mention that the clock was synchronized, which may refer to the use of an additional Brainproducts sync-box. I think it would be good to check and potentially explain this a bit more to avoid confusion. As I said before, the EEG data from inside the scanner looks good, implying that it was set up correctly, so that I assume that this is just about clarification.

Thanks for catching this ambiguity. We do use a Brain Products SyncBox during acquisition. This takes the 10 MHz MRI clock and produces a 5 kHz output signal to which the EEG acquisition is phase locked. We’ve corrected and added detail in the revised MS.

5) The thresholding approach is inconsistent, with cluster-forming thresholds being either p<.001 or p<.05, which is very different. The latter is also explicitly discouraged, if I am not mistaken: <https://pubmed.ncbi.nlm.nih.gov/27357684/>

It is important to point out that we consistently adopt a cluster-definition threshold of p < 0.001 for all analyses based on variance in MRI over space. When we adopt a cluster-definition threshold of p < 0.05, this is applied to temporal clusters in analysis of EEG data or EEG/MRI relationships.

As the reviewer points out, there is an issue in analysis of BOLD results that stems from poor understanding of the spatial autocorrelation of BOLD MRI. The software we use, like most, assumes a Gaussian function with fixed parameters defined by random field theory. As noted in Eklund, Nichols, and Knuttson (2016, PNAS), as well as in an earlier paper from the same group (Eklund et al., 2012, Neuroimage) and in other paper from other groups, the empirical function commonly has higher kurtosis than assumed. As a result, the probability of a single voxel observation reaching a chosen statistical threshold, given the null, will be larger than reflected in the nominal threshold value. Eklund et al. (2016) use simulation to demonstrate that treating a single-voxel result as potentially meaningful when it reaches a threshold of p < 0.01 inflates the emergence of significant clusters in subsequent permutation analysis. Woo, Krishnan, and Wager (2014, Neuroimage) use similar simulation approaches to show that a more conservative threshold of p < 0.001 addresses this issue, and this cluster-definition threshold has accordingly become widely used in the literature.

(It’s perhaps worth mentioning that in the specific context of our paper, this conservative threshold may be overkill because we do not interpret BOLD results. All of our results ultimately describe analysis of similarity - similarity of BOLD patterns in classification analysis, or correlation of classification results to EEG variance over permuted samples, or correlation of BOLD variance to EEG variance over permuted samples. To our knowledge and understanding, there is no reason to expect these measures to show the high-kurtosis spatial autocorrelation that emerges in BOLD. However, use of a more liberal threshold does not generate qualitatively different results and would likely reduce reader confidence, so we have stuck with p < 0.001.)

We use a cluster-definition threshold of p < 0.05 for analysis of temporal clusters in EEG classification results and in the relationship between EEG voltage and MRI. Neither of these types of analysis is dependent on assumptions regarding the spatial autocorrelation of MRI data. That’s self-evident in analysis of EEG classification. In EEG/MRI, this can be demonstrated from the observation that temporal analyses of EEG/MRI rely on similarity analysis of temporally fixed MRI results (in ROIs defined by searchlight analyses that employ a threshold-definition of p < 0.001). The variance of the EEG-to-MRI relationship over time is therefore generated entirely by variance in the signal derived from EEG. There is no opportunity for inaccurate estimation of MRI variance to bias the emergence of clusters over time simply because there is no variance in the MRI signal over time.

Reviewer #2: Summary:

This paper presents two experiments investigating the relationship between early attentional deployment and the categorization of objects that were spatially attended vs. not attended in a search-like display. Using a combination of ERP markers and fMRI data, this study presents a novel and interesting data set that in a first experiment establishes a relation between early attentional selection (marked by the N2pc component of the ERP) and object categorization using EEG classification data; in a second experiment, the N2pc is further associated with activity patterns in various brain regions. Overall, this study undoubtedly presents a rich and interesting new data set. At the same time, it's a bit unclear what the major theoretical contributions are and what some of the N2pc-to-brain regions links mean. I also had several clarification questions with regards to the analyses which were often described rather briefly. Below are my detailed comments and questions.

Also I want to note that the manuscript file was named _R1, so I assume I was brought in as a reviewer in the second round of revisions. However, I did not see the revision or response letter to the first round of revisions, so treating this like a new manuscript.

Our thanks to R2 for the insightful evaluation of the MS.

The editor notes in the decision letter that the MS was initially sent to a specialist academic editor for evaluation before being sent for standard review, and we expect this explains the ‘R1’ tag. This is the first set of reviews we’ve received.

Introduction/Motivation

1) I am not sure I agree that the "emergent [attention] model suggests that strategic selection begins with the derivation of schemas and templates from conceptual knowledge" (pg. 3). There is quite a good amount of attention research suggesting / arguing that low-level features and locations are what guides attention and selection occurs based on these lower-level representations that may feed into higher-level representations, but nonetheless play a critical role in how attention is allocated (For example see earlier TICS paper by Chapman et al. 2024 on these topics).

We agree that low level visual features play a role in how attention is deployed, and that there is a complicated interplay of features, locations, and strategy that defines how attention is deployed and maintained. Our point here is simply that selection of a target depends on knowledge of what constitutes a target.

This appears entirely in line with Chapman and Störmer (2024, TICS). That paper is concerned with the manner in which ‘relevance’ results in the prioritization of features, locations, and objects in the environment. It largely leaves aside the question of how ‘relevance’ is created, but the framework clearly rests on the idea that prior knowledge is involved.

2) A related point: "We presume that information selected through the deployment attention activates or constructs neural representations of concepts and categories" (pg., 3/4, abstract etc) — who is "we" in this context? Is this a reference to the field of attention researchers, or the present authors? Again, I wasn't sure what the evidence is for this statement and motivation here— it's not super clear that this is a common theory of what attention does.

Thanks for this. We’ve revised to reduce the use of a royal ‘we’.

Our intent here is to briefly convey that in naturalistic settings, attention is commonly employed to gather information about the environment. We cite two theoretical perspectives that place this function at the center of our understanding of attention. We can add additional citations in this context if the reviewer sees this as useful.

Methods/Results:

3) Why were the scrambled objects presented? I assume to match stimulus presentation across hemispheres, but as the authors allude to, the morphed vs. real object images are not perfectly matched.

Lateralized brain activity in the ERP is sensitive to imbalances in low level sensory characteristics. For example, if the left visual hemifield contains stimuli of higher contrast than the right hemifield, the evoked potentials observed over left and right visual cortex will not be equal. This is thought to reflect the fact that higher contrast in one hemifield drives a larger neural response in early contralateral cortex, and thus a ‘larger’ evoked response. It complicates interpretation of ERP components defined by laterality (Luck 2004).

To minimize the impact of this sensory difference, we tried to keep the search displays roughly equivalent in terms of the number, luminance, size, and color composition of objects presented in the left and right visual hemifields. At the same time, we did not want to present additional recognizable, real-world objects as non-targets. We were concerned that this would result in degraded performance in EEG and MRI classification of target and distractor categories.

Our approach was therefore to employ non-target stimuli that were morphed versions of our objects, and thus perfectly matched in many low-level features (such as total luminance, color composition, and size). We hoped that these objects would generate the same sort of contralateral sensory response as an example of a complete object but would not excessively confuse classification.

We were not totally successful in this. Our morphed objects differ from the complete objects in their spatial frequency and in the statistical properties of contour complexity and intersection. We believe that this drives laterality in the ERP results that precedes the N2pc, as described in the paper.

A better approach may be to use non-target stimuli that have all the same low-level properties as real-world objects. However, to our knowledge, there are no algorithmic approaches to the generation of such stimuli.

Also it says the objects were chosen to be "visually different". Was this verified using a model, e.g., CNNs, or was this decided visually by the authors?

The stimuli were informally selected by the authors to generate a visually heterogeneous set.

The use of CNNs for verification of stimuli heterogeneity between categories is somewhat problematic; ultimately the CNN will be sensitive to categorical definition despite visual heterogeneity, and this can emerge in early model layers. Use of the model for this sort of purpose therefore requires assumptions about the type of information captured within a given model layer.

That said, if we run our stimuli set through a CNN - eg. CORnet S – we find activations in early layers that are as similar across object categories as they are within categories. At the level of representation captured in these early model layers, the model does not appear to capture image properties that could be used to infer category membership.

Including this analysis in the paper would require a tangent in the narrative, with explanation of CNNs and their limitations for this purpose. We are happy to be guided by the reviewer on whether this is necessary.

4) Linking of EEG to fMRI data: I may have missed this, but how do the authors control for multiple comparisons given many brain sites they are testing for correlating with the EEG data?

It’s important to point out that the study was conducted with a clear *a priori* focus on the N2pc (as measured as mean voltage at its peak) as a predictor of MRI results. In this set of analyses, the EEG observation is fixed and the number of contrasts is equal to the number of voxels (or searchlights, which is equivalent). FWE is controlled using permutation analysis of voxel clusters defined in 3-d space.

In subsequent analyses, we flip this strategy, extracting MRI results from defined regions of interest and comparing to EEG data over time. In these analyses, the MRI data is fixed and the number of contrasts is equal to the number of EEG samples. FWE is again controlled using permutation analysis of clusters, in this case with clusters defined in 1-d time.

Our approach to FWE correction is detailed in the relevant subsection of the results section, or in the relevant subsection of the methods when derivation is more complicated.

5) Do I understand correctly that participants were asked to do a 4-way category discrimination, yet only used two response buttons such that 2 categories were mapped onto the same button? That seems a bit odd if the question is about categorization, as really what participants needed to do it group two categories to respond correctly?

That is correct. This approach was adopted to equate the motor response required by two separate object categories. We approached the experiment with the concern that category classification might load strongly on brain activity underlying preparation or execution of the associated response. By having two categories associated with the same response, we reduced the opportunity for this to occur and retained the ability to conduct 2-category classification analyses under circumstances where motor activity could not distinguish the categories.

We do not report results from these 2-category ‘sanity check’ analyses in the paper, but we are able to classify two object categories with high accuracy from both EEG and MRI when the response evoked by both categories is the same.

This does mean that participants likely grouped the two categories because of their shared response. However, our results demonstrate that the individual object categories continue to evoke activity that can be used to accurately reconstruct the object categories that shared a response, and that such categorization emerges in areas that are not commonly assumed to play a role in motoric response, such as ventral visual cortex.

We’ve provided more detail on this design characteristic on p.22 of the revised manuscript.

6) It says trials were not averaged prior to classification — but the "chunking" refers to averaging across small portions of trials, is that correct? I just want to make sure it's clear whether the classifier uses small subsets of averaged EEG data (ERPs) or single-trials.

For the purpose of classification, trials were not averaged. The chunks define which set of trials is employed for classifier training and which set for classifier testing, but trialwise data is submitted to the classifier, and classifier output reflects the percentage of trials that were correctly classified.

For the purpose of calculation of N2pc amplitude, trials were averaged.

We have revised content in the ‘Classification of EEG section’ section of the methods to make this clearer.

7) For the classification, the data is split into target-lateral and distractor-lateral trials, following the same logic of the N2pc component. Thus , if I understood correctly, training and testing data sets are different, which makes it somewhat difficult to interpret the classification accuracy of the EEG data (e.g., Fig. 2C).

Trials were indeed split into lateral target and lateral distractor conditions. But within each of these two visual conditions, the data was partitioned into training and testing sets for each cross-validation fold. The same data thus contributes to classifier training and testing across folds for each of the target-lateral and distractor-lateral conditions separately.

It could be that the difference is driven by differences in the training set due to various reasons (e.g., lower quality of the data overall, or some other variable that is different than the one of interest here).

We are not certain that we follow the reviewer’s idea here, but it may be that the suggestion is that target-lateral and distractor-lateral trials have structural differences that make comparison of classification accuracy in these conditions problematic.

Our experimental design was such that there was no structural difference in the search arrays containing a lateral target versus a lateral distractor. Exactly the same search arrays could emerge in target-lateral or distractor-lateral conditions, with only the preceding semantic cue distinguishing these conditions. There are the same number of target-lateral and distractor-lateral trials in the design.

Ideally, the training set would be "neutral" condition, and then testing would occur on attended and unattended objects. I realize that's not possible in the current task design, but have the authors tried to train on all the data and then test either on target- or distractor-lateralized activity?

We appreciate the suggestion to train on all stimuli and subsequently test on attended and unattended objects. We have not done this for two reasons. First, it has low power. Training on combined data will mean that targets can appear on the vertical or lateral locations, and this variability in location will introduce variability in signal that will degrade classifier performance. Second, this analysis tests a different hypothesis than that addressed by our current analyses. Combined training assumes that there are commonalities in the brain activity evoked by object categories when they act as targets and distractors, and that attention acts solely by enhancing or suppressing these commonalities. This is not necessarily the case. For example, attention may act to enhance information about a target, and suppress information about a distractor, without acting on the same representational features in these distinct operations.

8) Did the FMRI classification use a similar approach to train and test for lateral-target and lateral-distractor? It was not exactly clear to me how target vs distractor activity was extracted in this case, and whether laterality should matter for the different brain regions.

Unimodal fMRI classification uses all trials, ignoring location. We have clarified this in the revised text (p.25).

In the association of N2pc to MRI classification, MRI classification occurs separately for target-lateral and distractor-lateral, such that the same trials contribute both to calculation of N2pc amplitude and to calculation of classification accuracy.

9) The reported links between the N2pc and various brain regions is interesting, and the authors are doing a good job trying to walk through what these links could mean. Yet, I couldn't fully follow or understand the theoretical implications of the three main brain areas found: lateral PPC, insular, and VMPC. As the authors say themselves, these areas have been implicated in various functions and tasks, and so I was not convinced that this allows drawing the conclusion that this shows that spatial attention is directly linked to object categorization. Maybe the authors can be more careful here.

Importantly, our conclusion is not that spatial attention is directly linked to object categorization in the identified brain areas. Our interpretation is rather that spatial attention propagates information about object categories to the identified structures. We expect that categorization precedes the emergence of this information in insula and prefrontal cortex, and that cognitive operations in insula and prefrontal cortex depend on conceptual information about the target category. We have revised in several sections of the manuscript to make this clearer.

As the reviewer notes, it is hard to pin down how such abstracted information is used in these areas. We provide some possibilities in the discussion section, but this is very much conjecture, and we have revised to make this clear.

Minor:

10)When reading the manuscript and first looking at the ERP data, I immediately thought about the fact that the N2pc effect appears sustained (or N2pc followed by a CDA-like component); the authors later on report analyses across the longer time interval, but it might be nice to announce this earlier on.

We’ve added a preemptive line to the second paragraph of the results section of the revised MS.

Reviewer #3: The study aims to identify the temporal and anatomic characteristics of the propagation of attention-mediated conceptual information in the human brain. To address this question, the authors carry out two experiments, one unimodal EEG experiment, and a second experiment with concurrently-recorded EEG and fMRI, with a similar task performed by the participants in both experiments. The recorded data is valuable and provides the advantage of looking at both the temporal and spatial dimensions of attentional effects on semantic content formation in the brain. The authors perform several analysis steps, looking at the N2pc signal in EEG data, target category classification in fMRI data, and the relationship between these two signatures of attention using the concurrently-recorded EEG and fMRI data. They show the relationship between the target-elicited N2pc in EEG and the target classification accuracy in fMRI data. They further identify brain regions in which target

category information is predicted by target-elicited N2pc amplitude.

One strength of the study is the use of both EEG and fMRI data, providing the opportunity to look at the temporal dynamics in addition to providing a good spatial resolution.

Another strength is that they show the relationship between the target-elicited N2pc and fMRI data both at the univariate level (voxel activity) and at the multivariate level (target classification).

Identifying the brain regions that are suggested to play a role in the emergence of conceptual information is an important result.

The manuscript would benefit from addressing these points:

1- Considering the relationship between N2pc amplitude and fMRI target category classification accuracy, the authors draw a causal conclusion from a positive correlation (e.g. in page 9 in the last paragraph and page 12). While they provide the lack of a significant relationship between distractor-elicited N2pc and target information as justification, this is still not enough justification for a causal relationship. The authors should either tone down about the causal relationship throughout the manuscript, or provide further stronger justification.

Our thanks to R3 for the insightful evaluation of the MS.

We’ve revised the relevant passages to make clear that the results are correlational, explicitly stating that causation is not demonstrated.

2- Page 14, section on "Coupling variance in lateral EEG tovariance in fMRI-derived category information":

Some discussion is needed on why there is difference between the three ROIs in the latency of significant correlation between lateral EEG and fMRI category information. The section talks about the results of the correlation and concludes in a sentence that these results suggest a close link between selective processes in N2pc and the propagation of target-category information to these areas. However, the results in Figure 6 show that the relationship between EEG laterality and target category information emerges at different times with different durations in the three ROIs. These differences should be addressed more explicitly. It should also be discussed whether this difference affects the conclusion.

This section focuses on results observed in the IPL cluster, as category information in this area is predicted by lateral EEG amplitude over a long interval. We suggest some account for why this could be the case. In the revised manuscript, we’ve added a line to explain that these mechanisms would act in concert with those reflected in N2pc to guide information gathering.

The relationship between lateral EEG and category information for the insula and VMPFC is quite discrete to the latency of the N2pc (Fig 6a, 6b). There are small differences in terms of onset and duration of the significant cluster, but these are not likely to be statistically reliable.

3- The authors should address the difference in the ERP and classification results of the two EEG experiments (Fig. 2). This includes the differences observed in the lateral occipital voltage (Fig. 2a and 2d) and contralateral-ipsilateral voltage (Fig. 2b and 2e), as well as in EEG classification accuracy (Fig. 2c and 2f).

The circumstances of the experiments differ quite dramatically - in one case the subject is seated in a comfortable chair in a quiet room, viewing a computer screen with larger stimuli presented across a wider field of view. In the other they are supine in a noisy scanner, viewing a projected image via a mirror mounted to the visible head coil, and stimuli are smaller and presented across a narrower field of view. Scanning requires that the participant be completely still, and it is common for participants to become uncomfortable, particularly when the electrode cap is fitted. Both these differences in physical stimulation and in participant state will impact the EEG data. We’ve added a note to this effect to the revised MS. In spite of all this, the data is similar across the experiments. We have not tested for significant differences across the experiments, as we had no a priori expectation of such, but we do not expect that any would reach statistical significance.

4- -Details about stats are missing in some parts (e.g. p. 16, second paragraph)

The statistical results provided in this section of the results, which is titled ‘Coupling variance in EEG indices of attentional selection to variance in fMRI voxel activity’, are derived using the procedure described in the corresponding portion of the methods. This section had the title ‘Coupling variance in N2pc to variance in MRI voxel activity’ in the original MS; that’s been revised in the new MS so that the section titles are identical.

5- Figure 2 caption b and e: should be "contralateral minus ipsilateral" instead of "ipsilateral minus contralateral"

Thanks for catching this. Corrected.

6- References 27 and 48 are the same, also references 15 and 26.

Corrected.

7- Reference 37 is under review. Maybe provide the information of the bioRXiv version?

Thanks for the suggestion; we’ve made this change.
