# Supplementary material for: Neural mechanisms for the attention-mediated propagation of conceptual information in the human brain
Source: PLoS Biol. 2025 Mar 28;23(3):e3003018. doi: 10.1371/journal.pbio.3003018 (PMC12135929; doi:10.1371/journal.pbio.3003018)
Supplement: Attachment — Submitted filename: Acunzo et al - round 2 response to reviewers.docx [file pbio.3003018.s004.docx]

Acunzo, Grignolio, & Hickey

Neural mechanisms for the attention-mediated propagation of conceptual information in the human brain

Response to editor and reviewers

**Editor remarks:**

*We would like to suggest a different title to improve its accessibility for our broad audience: "Neural mechanisms for the attention-mediated propagation of conceptual information in the human brain"*

Done.

*Please add the links to the funding agencies in the Financial Disclosure statement in the manuscript details.*

Done.

*Please include information in the Methods section whether the study has been conducted according to the principles expressed in the Declaration of Helsinki.*

Done.

*Deposition in a publicly available repository. Please also provide the accession code or a reviewer link so that we may view your data before publication.*

The data will be posted at the University of Birmingham edata repository, which is an institutional repository designed to fulfill UK and EU funding agency requirements. The data will be available at this link:

edata.bham.ac.uk/id/user/595

We are in the process of curating and organizing data for post at this site, and all required material will be at this location by the end of January 2025.

*Please ensure that your Data Statement in the submission system accurately describes where your data can be found.*

Done.

*Per journal policy, if you have generated any custom code during the course of this investigation, please make it available without restrictions.*

All code for analysis and generation of raw figures will be available at the link provided above. We are in the process of curating and organizing code for post at this site, and this will be available by the end of January 2025.

**Reviewer remarks:**

 *Reviewer #1: The authors have undertaken a thorough and thoughtful revision. All of my points were addressed.

That being said, personally, I would add the topo plot of the N2pc difference from within the scanner to the manuscript (#2 of my review), and provide a short version of the explanation for the more complex voltage distribution as a footnote. I think this is useful information, and also illustrates some of the complexity of running MR-EEG experiments. In my mind, it also does not undermine the inferences of the paper at all. In case you end up adding it, make sure to check again whether it is a left-minus-right or right-minus-left difference. This was inconsistent across the caption you provided and the text.*

Our thanks to the reviewer for the evaluation and feedback. We’ve adopted an in-between approach here; we’ve included scripts and images in the code and data package that generates and described the topography of the N2pc. We also include notes in that package regarding the possible impact of scanner artefacts on frontal electrode signal. This information is therefore available to the invested reader who takes a deep dive into the data and methods. At the same time, this approach reduces the likelihood that a less-invested reader becomes confused or discounts the results because of the messy whole-head topography.

*Reviewer #2: The authors have addressed my questions in their response letter.*

Our thanks to the reviewer for the evaluation and feedback.
